# Evaluation of the IKKβ Binding of Indicaxanthin by Induced-Fit Docking, Binding Pose Metadynamics, and Molecular Dynamics

**DOI:** 10.3389/fphar.2021.701568

**Published:** 2021-09-10

**Authors:** Mario Allegra, Marco Tutone, Luisa Tesoriere, Alessandro Attanzio, Giulia Culletta, Anna Maria Almerico

**Affiliations:** Dipartimento di Scienze e Tecnologie Biologiche Chimiche e Farmaceutiche (STEBICEF), Università degli Studi di Palermo, Palermo, Italy

**Keywords:** indicaxanthin, IKKβ, induced fit docking, molecular dynamics, anticancer activity, binding pose metadynamics

## Abstract

**Background:** Indicaxanthin, a betaxanthin belonging to the betalain class of compounds, has been recently demonstrated to exert significant antiproliferative effects inducing apoptosis of human melanoma cells through the inhibition of NF-κB as the predominant pathway. Specifically, Indicaxanthin inhibited IκBα degradation in A375 cells. In resting cells, NF-κB is arrested in the cytoplasm by binding to its inhibitor protein IκBα. Upon stimulation, IκBα is phosphorylated by the IKK complex, and degraded by the proteasome, liberating free NF-κB into the nucleus to initiate target gene transcription. Inhibition of the IKK complex leads to the arrest of the NF-κB pathway.

**Methods:** To acquire details at the molecular level of Indicaxanthin’s inhibitory activity against hIKKβ, molecular modeling and simulation techniques including induced-fit docking (IFD), binding pose metadynamics (BPMD), molecular dynamics simulations, and MM-GBSA (molecular mechanics-generalized Born surface area continuum solvation) have been performed.

**Results:** The computational calculations performed on the active and inactive form, and the allosteric binding site of hIKKβ, revealed that Indicaxanthin inhibits prevalently the active form of the hIKKβ. MM-GBSA computations provide further evidence of Indicaxanthin’s stability inside the active binding pocket with a binding free energy of −22.2 ± 4.3 kcal/mol with respect to the inactive binding pocket with a binding free energy of −20.7 ± 4.7 kcal/mol. BPMD and MD simulation revealed that Indicaxanthin is likely not an allosteric inhibitor of hIKKβ.

**Conclusion:** As a whole, these in silico pieces of evidence show that Indicaxanthin can inhibit the active form of the hIKKβ adding novel mechanistic insights on its recently discovered ability to impair NF-κB signaling in melanoma A375 cells. Moreover, our results suggest the phytochemical as a new lead compound for novel, more potent IKKβ inhibitors to be employed in the treatment of cancer and inflammation-related conditions.

## Introduction

**FIGURE 1:**
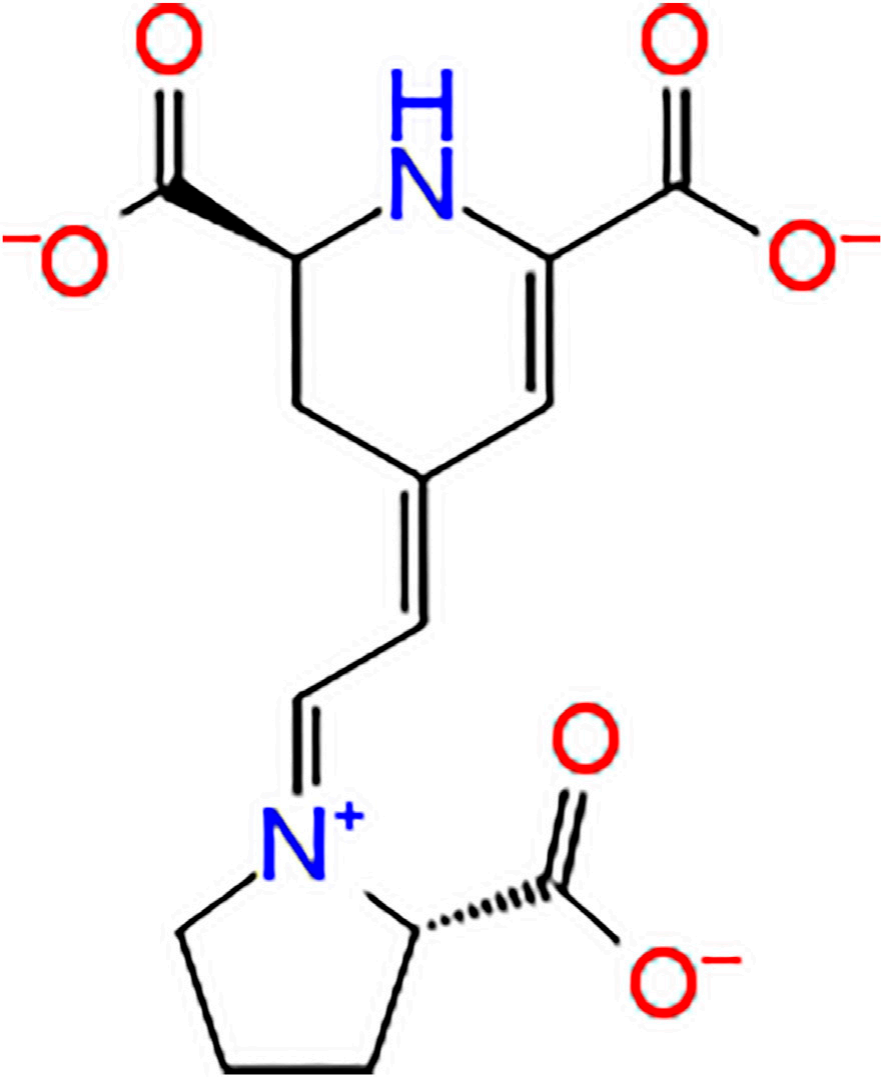
Indicaxanthin structure.

Indicaxanthin, ((2S)-2,3-dihydro-4-[2-[(2S)-2-carboxypyrrolidin-1-yl]ethenyl]pyridine-2,6 dicarboxylic acid), is a betalain pigment belonging to the betalain class compounds (Figure 1). This includes vacuole pigments restricted to fruits and flowers of 10 families of Cariophyllalae plants and a few superior fungi of the genus Amanita of the Basidiomycetes (Slimen et al., 2017). Indicaxanthin has been investigated over the last 20 years by the author’s research group for its chemical and nutraceutical properties. Interestingly the phytochemical has been demonstrated to be highly bioavailable in humans and even permeable to the rat brain-blood-barrier (Tesoriere et al., 2004; Allegra et al., 2015). Moreover, because of its reducing and amphipathic properties, it has been shown to interfere with cellular, redox-dependent signal transduction pathways in several experimental models of inflammatory-related, oxidative stress-dependent pathological conditions. Along these lines, Indicaxanthin has been demonstrated to exert significant reducing, anti-oxidative, anti-inflammatory, spasmolytic, and neuromodulatory effects both *in vitro* and *in vivo* (Allegra et al., 2019)*.*

In addition to its redox-modulating and anti-inflammatory properties, Indicaxanthin has also been shown to exert antiproliferative effects against melanoma cells being also able to significantly impair tumor progression in a mouse model of cutaneous melanoma (Allegra et al., 2018). Mechanistic evaluations have individuated in the inhibition of the nuclear factor-κB (NF-κB) signaling, a key event underlying the antitumoral effects exerted by the pigment.

The NF-κB transcription factor family consists of five different DNA-binding proteins that form a variety of homodimers and heterodimers regulating both the innate and adaptive immune responses (Taniguchi and Karin, 2018). More interestingly, besides its modulating effects on the entire inflammatory response, NF-κB is responsible for accelerating cancer progression, metastasis, angiogenesis, and drug resistance (Zhang et al., 2017). Indeed, in several types of cancer, both in malignant cells and in the tumor microenvironment, NF-κB is constitutively activated, and only rarely is such activation due to NF-κB-related genetic alterations (Staudt, 2010; Ben-Neriah and Karin, 2011).

Melanoma is an aggressive skin cancer, notoriously resistant to current cancer therapies (Van Herck et al., 2021). To effectively prevent chemoresistance development and reduce the risk of unwanted side effects, current pharmacological strategies employ a multi-targeted approach. Given that constitutive or drug-induced upregulation of NF-κB activity is associated with chemoresistance, NF-κB is considered one of the most relevant targets to discover new active compounds (Taniguchi and Karin, 2018). Permanent activation of NF-κB signaling in melanoma has been reported to proceed through the activation of the so-called canonical pathway (Yang and Richmond, 2001; Dhawan and Richmond, 2002; Amiri and Richmond, 2005; Amschler et al., 2010). Through this process, activation of NF-κB depends on the degradation of its specific inhibitors (IκB) consisting of IκBα, IκBβ, and IκBε. Typically, IκBs bind to NF-κB complexes, inhibiting their DNA binding and keeping them in a predominantly, inactive cytoplasmic form. Tumour microenvironmental stimuli can lead to the activation of a large cytoplasmic protein complex: the IκB kinase (IKK). The precise nature of this molecular mechanism remains to be elucidated but it contains IKKα, IKKβ, and IKKγ as the three seminal components. The phosphorylated and thus activated IKK complex is responsible for the phosphorylation of IκB, marking it for degradation through the proteasomal degradation machinery. The free NF-κB dimers (p50-p65) can, then, translocate from the cytoplasm to the nucleus, bind to DNA, and regulate gene transcription. Typical targets within the classical NF-κB signaling include genes encoding pro-inflammatory cytokines, growth factors, chemokines, matrix metalloproteinases, pro-proliferative proteins, anti-apoptotic proteins, pro-inflammatory enzymes, angiogenic factors, and adhesion molecules (Staudt, 2010). As a primary druggable mediator of canonical NF-κB signaling, the IKKβ enzyme inhibition has been the historical focus of drug development pipelines. Thousands of compounds with activity against IKKβ have been characterized, with much demonstrating promising efficacy in pre-clinical models of cancer. However, severe on-target toxicities and other safety concerns associated with systemic IKKβ inhibition have so far prevented the clinal approval of any IKKβ inhibitors (Prescott and Cook, 2018).

Indicaxanthin has been demonstrated to inhibit IκBα degradation in melanoma A375 cells at 100 μM, a concentration at which it impairs NF-κB signaling and inhibits 50% cell proliferation (IC_50_) (Allegra et al., 2018). In the light of this evidence, we employed an *in silico* approach to evaluate Indicaxanthin’s inhibitory activity against IKK. To this end, molecular modeling and simulation techniques including induced-fit docking (IFD), binding pose metadynamics (BPMD), molecular dynamics simulations, and MM-GBSA (molecular mechanics-generalized Born surface area continuum solvation) free energy calculation have been performed.

## Materials and Methods

### Protein and Ligand Preparation

For the purpose study, the 2.83 Å resolution crystal structure of human IKKβ (hIKKβ), which is partially phosphorylated and bound to the staurosporine analog K252a (PDB ID: 4KIK) (Liu et al., 2013) was used. The structure was optimized using the Protein Preparation Wizard in Maestro (Schrödinger, 2017) adding bond orders and hydrogen atoms to the crystal structure using the OPLS3 force field. Prime was used to fix missing residues or atoms in the protein and to remove co-crystallized water molecules. PROPKA was used to check for the protonation state of ionizable protein groups (pH = 7.0). The hydrogen bonds were optimized through the reorientation of hydroxyl bonds, thiol groups, and amide groups. In the end, the system was minimized with the value of convergence of the RMSD of 0.3 Å. Indicaxanthin and staurosporine analog K252a were prepared using LigPrep The force field adopted was OPLS3 (Jorgensen et al., 1996) and Epik 3.9 (Schrödinger, 2017-1) was selected as an ionization tool at pH 7.2 ± 0.2. Tautomers generation was unflagged and the maximum number of conformers generated was set at 32.

### Induced-Fit Docking

The induced-fit docking (IFD) is a method for modeling the conformational changes induced by ligand binding developed by Schrödinger (Sherman et al., 2006). This protocol models induced-fit docking of one or more ligands using the steps as also reported in (Tutone et al., 2019). Initial docking of each ligand is performed using a softened potential (van der Waals radii scaling). Then, a side-chain prediction within a given distance of any ligand pose is performed. Subsequently, a minimization of the same set of residues and the ligand for each protein/ligand complex pose is performed. After this stage, any receptor structure in each pose reflects an induced fit to the ligand structure and conformation. Finally, the ligand is rigorously docked, using Glide XP, into the induced-fit receptor structure. The grid boxes for the binding sites of Chain A (inactive form) and B (active form) were built considering the co-crystallized ligand staurosporine analog K252a as a centroid. For the allosteric sites of Chain A, the amino acid residues previously identified by Liu et al. (2018) were considered crucial for centering the docking grid. During the initial docking procedure, the van der Waals scaling factor was set at 0.5 for both receptor and ligand. The Prime refinement step was set on side chains of residues within 5 Å of the ligand. For each ligand docked, a maximum of 20 poses was retained to be then redocked at XP mode.

### Binding Pose Metadynamics

Binding pose Metadynamics (BPMD) is an automated, enhanced sampling, metadynamics-based protocol. During the simulations, the ligands are forced to stir in their binding pose. This method showed the ability to reliably discriminate between the correct ligand binding pose and plausible alternative generate with docking or induced-fit docking studies (Clark et al., 2016).

Ten independent metadynamics simulations of 10 ns each were performed using as a collective variable (CV) the measure of the root-mean-square deviation (RMSD) of the ligand heavy atoms concerning their starting position. The alignment before the RMSD calculation was done by selecting protein residues within 3 Å of the ligand. The alpha carbons of these binding site residues were then aligned to those of the first frame of the metadynamics trajectory before calculating the heavy atom RMSD to the ligand conformation in the first frame. The hill height and width were set to 0.05 kcal/mol (about 1/10 of the characteristic thermal energy of the system, kBT) and 0.02 Å, respectively (Clark et al., 2016). Before the metadynamics run, the system was solvated in a box of SPC water molecules followed by several minimizations and restrained MD steps that allow the system to reach slowly the desired temperature of 300 K as well as releasing any bad contacts and/or strain in the initial starting structure. The final snapshot of the short unbiased MD simulation of 0.5 ns is then used as the reference for the following metadynamics production phase. The pivotal idea of BPMD is that ligands that are not stably bound to the target binding pocket will move forward higher RMSD as compared to the stably bound ones if they are exposed to the same biasing force. After the simulation, the stability of the ligand during the course is represented by three scores: PoseScore, PersistenceScore (PersScore), and CompositeScore (CompScore). PoseScore is indicative of the average RMSD from the starting pose. The steepest increase of this value is a symptom that the ligand is not in a well-defined energy minimum and probably it might not have been accurately modeled. PersScore is a measure of the hydrogen bond persistence calculated in the last 2ns of the simulation that have the same number of hydrogen bonds as the input structure, averaged over all the 10 repeated simulations. PersScore covers a range between 0 and 1, where 0 indicates that either the starting ligand pose did not have any interaction with the target or that the interactions have been lost during the simulations, while 1 indicates that the interactions between the staring ligand pose and the last 2 ns of the simulations have been retained. CompositeScore is the linear combination of PoseScore and PersScore, lower values equate to more stable complexes. Each complex, previously obtained, was run on Desmond on a single node with 1 GPU card, taking for a typical system (1 complex = 1 × 10 metadynamics run) 72 h.

### MD Simulations

The plain MD simulations were carried out using Desmond 4.9 (Bowers et al., 2007) using the OPLS3 force field (Jorgensen et al., 1996). The complexes were solvated in orthorhombic boxes using the TIP3P water model. Ions were added to neutralize charges. The systems were minimized and equilibrated at a temperature of 303.15 K and a pressure of 1.013 bar. The system was simulated as an NPT ensemble; a Nose–Hover thermostat and Martyna–Tobia–Klein barostat were used. The integration time step was chosen to be 2 fs. To keep the hydrogen–heavy atom bonds rigid, the SHAKE algorithm was used. A 9 Å cutoff radius was set for the short-range Coulomb interactions, and smooth particle mesh Ewald was used for the long-range interactions. For each system, we carried out 100 ns MD, with 1.2 ps detection ranges for energy, and 4.8 ps for the trajectory frames. Visualization and analysis of the MD trajectories were performed using Desmond simulation analysis tools in Maestro.

### MM-GBSA Binding Free Energy Calculation

The MM-GBSA approach employs molecular mechanics, the generalized Born model, and the solvent accessibility method to determine free energies from structural information circumventing the computational complexity of free energy simulations wherein the net free energy is treated as a sum of a comprehensive set of individual energy components, each with a physical basis (Genheden and Ryde, 2015). We applied this method to the snapshots extracted from the 100 ns production MD trajectories. Protein-ligand binding free energy using MM-GBSA was calculated as the difference between the energy of the bound complex and the energy of the unbound protein and ligand. In this work MM-GBSA calculations were also achieved in Prime software (Jacobson et al., 2004); the entropy term ‒TΔS was not calculated to reduce computational time. The VSGB solvation model was chosen using OPLS3 Force Field with minimized sampling method.

## Results and Discussion

The hIKKβ protomer adopts a trimodular structure that closely resembles that of *Xenopus laevis* (xIKKβ): an N-terminal kinase domain (KD), a central ubiquitin-like domain (ULD), and a C-terminal scaffold/dimerization domain (SDD). In particular, the selected crystal structure has one protomer in the active conformation with phosphorylated Ser177 and Ser181 (Chain B), and the other protomer is in the inactive conformation with the same residues of serine unphosphorylated in the activation loop (Chain A). Recently, Liu and co. identified a druggable allosteric site between KD and ULD.

**FIGURE 2:**
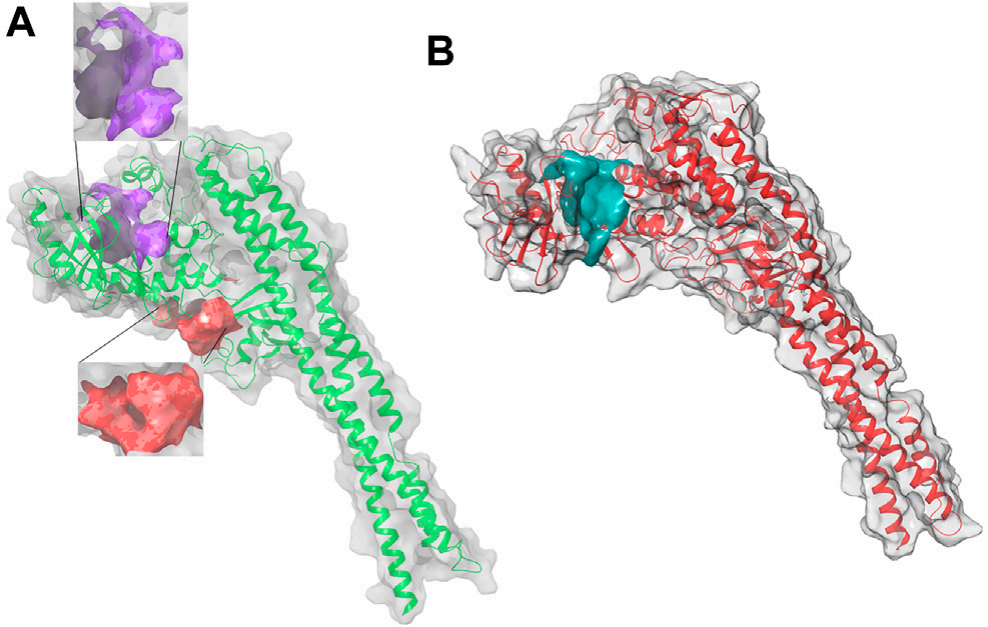
3D structure of hIKK forms: **(A)** the inactive form, Chain A, in purple the KD binding pocket, in red the allosteric binding site; **(B)** the active form, Chain B, in cyan the KD binding pocket of the inactive Chain A with a volume of 551 Å. This pocket is surrounded by three α helixes (Arg118-Ser127, Leu265-Leu273, and Leu303-His313) of KD and a loop (Thr368-Leu386) between two β sheets of ULD. They suggested that small molecules binding in the pocket between KD and ULD probably interfere with the kinase function by disrupting the interaction between these two domains (Liu et al., 2018).

To evaluate the binding capability of Indicaxanthin into the hIKKβ, we performed a series of computational studies with increasing accuracy, induced-fit docking (IFD), binding pose metadynamics (BPMD), unbiased molecular dynamics (MD) followed by MM-GBSA (molecular mechanics-generalized Born surface area continuum solvation) free energy calculation. We began the computational studies by selecting the crystal structure of the human IKKβ (PDB ID: 4KIK) (Liu et al., 2013) bound to the staurosporine analog K252a in the KD (Figures 2A,B). Firstly, we optimized the crystal structure by completing and refining the missing loops and residues and optimizing amide groups of asparagine (Asn) and glutamine (Gln), and the imidazole ring in histidine (His); and predicting protonation states of histidine, His, aspartic acid (Asp) and glutamic acid (Glu) and tautomeric states of histidine. Then, the docking studies have been performed centering the docking boxes on the 3D coordinates of K252a, both in the inactive Chain A and in the active Chain B. The RMSD of K252a in Chain B and A was calculated showing values 0.8–0.18 Å respectively (The superimposition of re-docked structures are reported in Supplementary Material). Moreover, another docking box for the allosteric binding pocket was centered on the previously identified residues by Liu and Co. (Liu et al., 2018).

### Induced Fit Docking

Induced-Fit docking (IFD) experiments were first carried out. IFD confers flexibility to the protein side chains, allowing the ligand to adjust and optimize binding interactions within the active site. We performed the IFD of K252a and Indicaxanthin in the two sites previously identified, and the IFD of Indicaxanthin in the allosteric binding site.

In most kinases, the ATP binding site is a narrow hydrophobic pocket located between the N-lobe and C-lobe of the kinase domain (KD) which are linked by a flexible hinge region. This binding site is partly covered by an activation loop comprised of Serine, Threonine, and Tyrosine residues in the unphosphorylated state. The N-terminal side of the activation loop consisting of a highly conserved triplet DLG (Asp166, Leu167, and Gly168) which is involved in the catalytic transfer of the γ-phosphate group (Xu et al., 2011). In IKKβ the residues Glu97, Tyr98, and Cys99 are part of the hinge region. The backbone groups of Glu97 and Cys99 can provide hydrogen bonding interactions with an inhibitor. (Leung et al., 2013).

**FIGURE 3:**
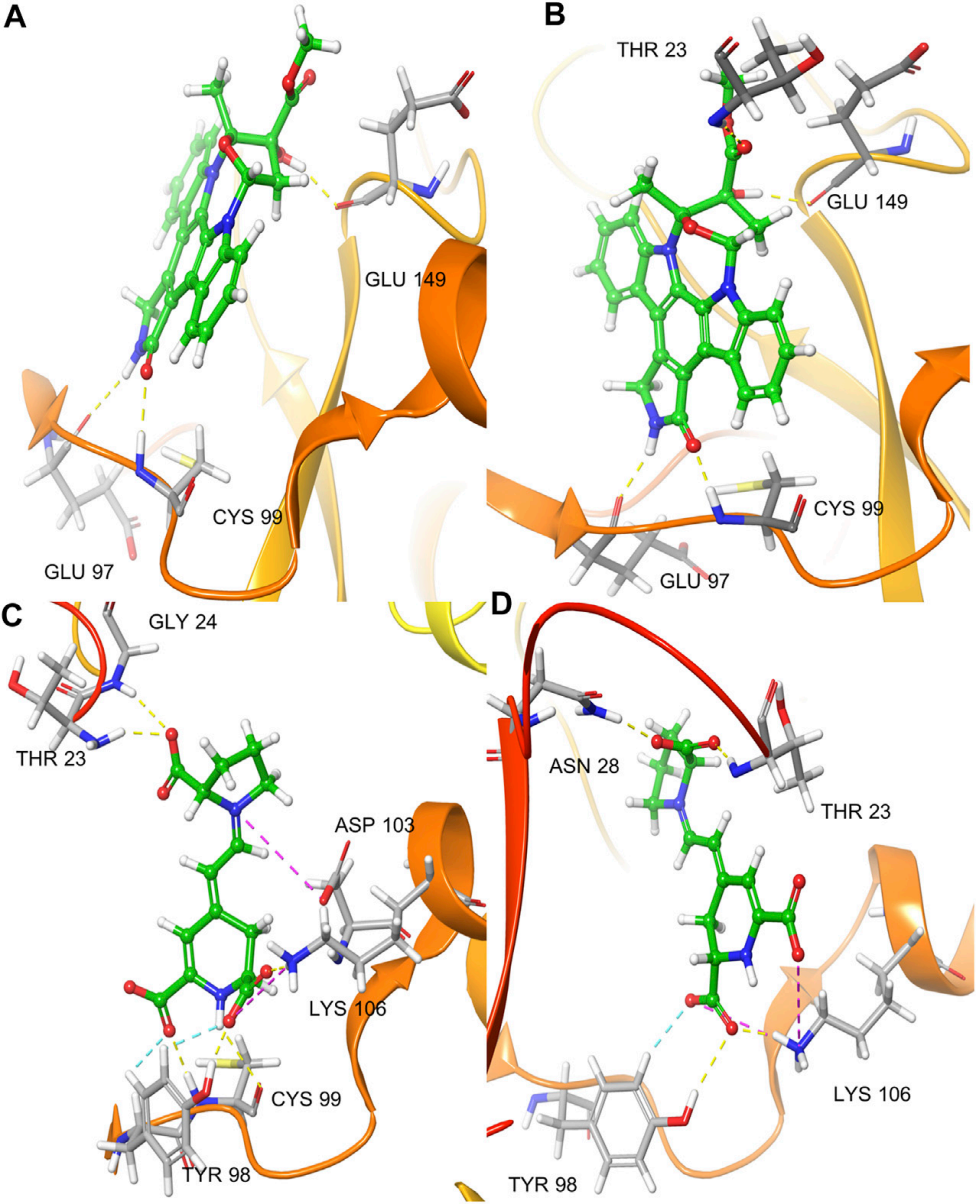
IFD pose of K252a in the inactive Chain A **(A)**; IFD pose of K252a in the active Chain B **(B)**; IFD pose of Indicaxanthin in the inactive Chain A **(C)**; IFD pose of Indicaxanthin in the active Chain B **(D)**. H-bonds interactions are represented in yellow dashes, aromatic H-bonds are represented in light blue dashes, the salt bridges in purple dashes.

Docking of K252a in Chain A showed a single H-bond interaction with Cys99 (hinge region), with the carbonyl of lactam ring. Other H-bonds were found between the amine of the K252a lactam group and Glu97 and the hydroxyl group and Glu149. Moreover, an aromatic H-bond was found with Asp166 (DLG triad). In chain B, K252a, as in Chain A, established the same interactions with Glu97, Cys99 (hinge region), and Glu149, with the only difference in another H-bond interaction between the carbonyl group of ether group and Thr23, and an aromatic H-bond with Tyr98. (Figures 3A,B).

**FIGURE 4:**
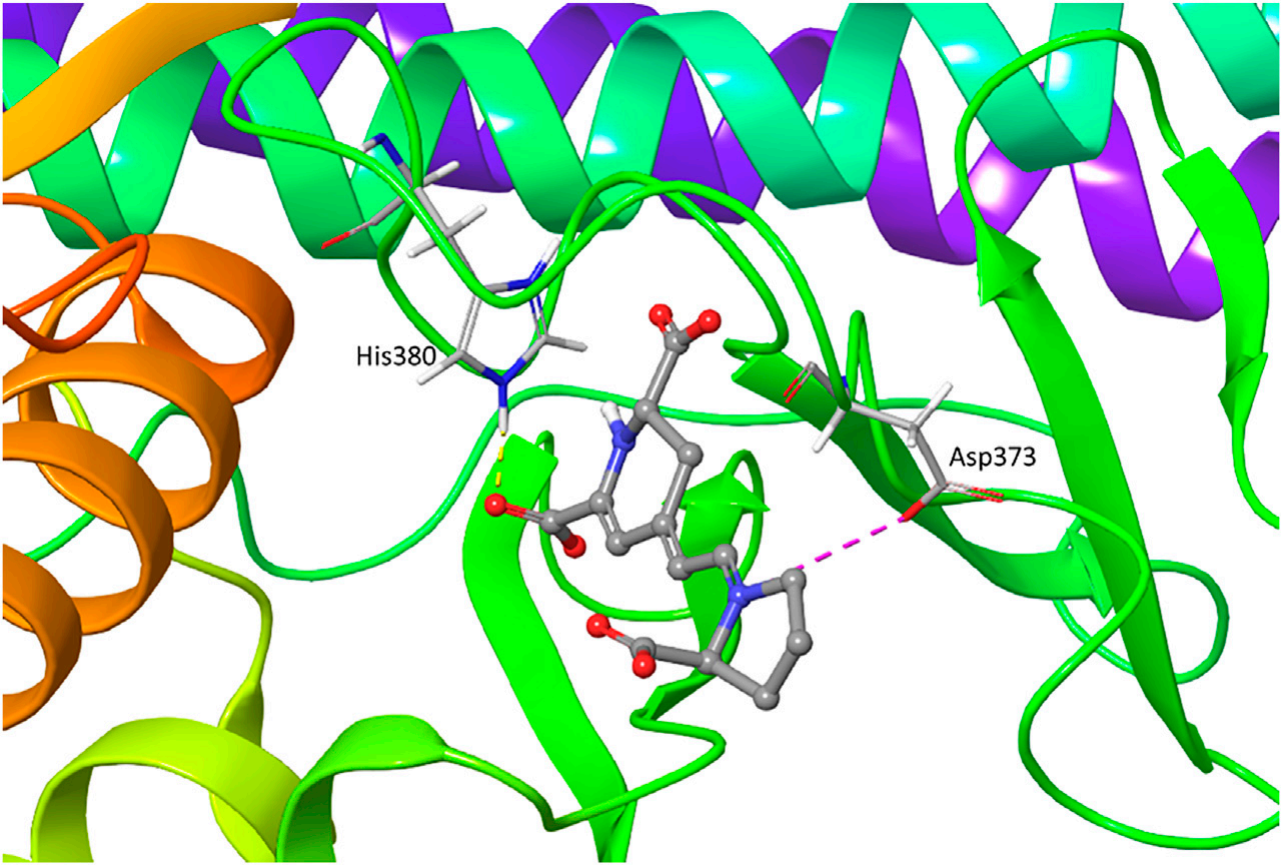
IFD pose of Indicaxanthin in the inactive Chain A allosteric binding site. H-bonds interactions are represented in yellow dashes, the salt bridges in purple dashes.

We performed the IFD of Indicaxanthin in the three sites identified previously. In the best IFD result, in particular, considering the Chain A (Docking score = −6.166, Table 1), the carboxyl group of the pyrrolidinium ring interacts with Thr23 and Gly24 forming two H-bonds, while the Cys99 simultaneously interacts with the carboxyl groups and the nitrogen of pyridine moiety. The carboxyl group of pyridine in position 11 interacts with Tyr98 (hinge region) with another H-bond and with Lys106 (solvent accessible region) through one salt bridge. The carboxyl groups in the pyridine moiety establish two aromatic H-bonds with Tyr98. Finally, another salt bridge involves the pyrrolidinium nitrogen and the Asp103. In Chain B (Docking score = −7.293), Indicaxanthin showed similar interactions as in Chain A. Two H-bond interactions involve the carboxyl group of the pyrrolidinium ring and Thr23, and Asn28; one of the carboxyl groups of pyridine interacts through H-bond with Tyr98, Lys106. The last interactions were a salt bridge between two carboxyl groups of the pyridine and Lys106 (Figures 3C,D). The IFD study of Indicaxanthin in the allosteric site (Docking score = −6.117) showed a single H-bond interaction was found between one of the carboxyl group of pyridine and His380; another interaction was a salt bridge, between the nitrogen of pyrrolidinium moiety and Asp373 (Figure 4).

**TABLE 1:** BPMD and docking scores for the Indicaxanthin and K252a complexes.

	Docking score	Pose Score	Pers Score	Comp Score
Indi/Chain A	−6.166	3.133	0.12	2.53
Indi/Chain B	−7.293	4.631	0.04	4.44
K252a/Chain A	−13.700	1.206	0.81	−2.85
K252a/Chain B	−14.121	0.909	0.64	−2.28
Indi/Chain A allosteric	−6.117	>6	0.0	>6

Moreover, we performed docking simulations with different hIKKβ inhibitors to be considered as reference compounds for the analyses of the binding interactions with key residues from the binding sites and to compare with the predicted binding affinity for Indicaxanthin. We selected three known orthosteric inhibitors: MLN120B (IC_50_ = 60 nM) (Prescott and Cook, 2018); the imidazo [1,2-a]quinaxoline derivative 6a (IC_50_ = 324 nM) (Moarbess et al., 2016); LASSBio-1524 (IC_50_ = 20 µM) (Guedes et al., 2016); and one allosteric inhibitor: BMS345521 (IC_50_ = 300 nM) (Prescott and Cook, 2018). The docking scores of these known inhibitors are consistent with the experimental IC_50_, considering that Indicaxanthin showed an IC_50_ = 100 µM. Additionally, the key residues identified for these known inhibitors are similar to those identified for Indicaxanthin (Cys99, Asp103, Lys106). The allosteric inhibitor BMS345521 showed key interactions with His380, already identified for indicaxanthin, Asn308, Ser127. The 2D and 3D figures of the docked reference inhibitors together with key interactions and docking scores are reported in Supplementary Material.

### Binding Pose Metadynamics

Binding pose metadynamics (BPMD) is an automated, enhanced sampling, metadynamics-based protocol, in which the ligand is forced to move around its binding pose. This method showed the ability to reliably discriminate between the correct ligand binding pose and plausible alternative generate with docking studies. In particular, Clark and colleagues introduced this metadynamics plus IFD strategy for accurate and reliable prediction of the structures of protein−ligand complexes at a useful computational cost. Their strategy allows treating the problem in full atomistic details, significantly enhancing the predictive power of IFD methods (Clark et al., 2016).

Firstly, we decided to use BPMD to evaluate the stability of the K252a best poses (in terms of docking scores) obtained from IFD studies into the binding site in Chain A and Chain B to evaluate the reliability of the IFD poses obtained as validation of the docking scoring functions. The results were defined in terms of pose stability based on the PoseScore that is the RMSD of the ligand related to the starting coordinates of the heavy atoms of the ligand. A PoseScore <2 Å is considered stable for the co-crystallized ligand. Moreover, another metric is used to evaluate the results such as the PersScore that is a clue of H-bonding formed between the ligand and the target during the simulations. In general, maintaining 60% of the total H-bonds (PersScore>0.6) is considered a good sign of stable interaction. The linear combination of these two scores provides a third score, the CompScore which is calculated as follows:

CompScore = PoseScore—5 × PersScore.

Lower values of this equate indicate more stable complexes.

The simulation performed on K252a pose in Chain A showed a PoseScore of 1.206. The PersScore showed that the hydrogen bonds were kept for 81% of the simulation time. The interactions by the lactam ring were confirmed to stabilize the molecule, in particular, we observed for 98.2% of the simulation time, interactions between the amine group and Glu97, for 97.3% of the simulation time interactions between the carbonyl oxygen and Cys99. The H-bond interaction between the hydroxyl group and Glu149 was kept for 48.2% of the simulation time. The CompScore of −2.852 confirmed that the starting molecule pose is stable into the active site.

The results of the K252a pose in Chain B showed a PoseScore of 0.909, the PersScore proves that for 63.9% of the simulation time, the H-bonds were maintained. As in Chain A, the interactions by the lactam ring were confirmed, in particular, 100% of the simulation time, the interaction between the carbonyl oxygen and Cys99, and 98.2% of the simulation time interaction between the amine group and Glu97. The same for H-bond interaction between the hydroxyl group and Glu149 was kept for 57.3% of the time. The linear combination of PoseScore and PersScore, CompScore, was −2.284. The result obtained confirmed the accuracy of the identified IFD poses. (Table 1).

Successively the BPDM simulations have been performed at the binding site in Chain A, the best pose of Indicaxanthin reached a steady PoseScore of 3.133, considered stable, while PersScore showed that the hydrogen bonds identified at the start of the metadynamics run were kept for 12% of the averaged time. In particular, the H-bond between carboxyl oxygen and Cys99. The CompScore value was 2.533.

At the binding site in chain B, the averaged RMSD of Indicaxanthin reached a steady PoseScore of 4.631, PersScore of 0.04. In particular, three H-bond were recorded during the ten replicas. H-bond interaction between the carboxyl group of pyrrolidinium moiety and Thr23 was kept for 11.8% of the simulation time, the same group showed an ulterior H-bond with Asn28 for 7.3% of the simulation time. The last interaction was between one of the carboxyl groups of pyrimidine and Lys106 for 2.7% of the simulation time. This interaction was supported by salt bridges between the two inferior carboxyl groups and Lys106. The last score, CompScore was 4.441. The BPMD analysis confirmed that the poses obtained from the IFD are valuable for Indicaxanthin and they can be used to perform a time-dependent study which could give more insights about the binding of Indicaxanthin. The BPDM results of Indicaxanthin into the allosteric binding site give unstable values suggesting that Indicaxanthin is not a stable binder of the allosteric site. However, to gain more insights, we decided to perform also the time-dependent simulation of this latter complex. (Table 1).

### Molecular Dynamics Simulations

The running of dynamics simulations of protein-ligand complexes over time could be considered the major accuracy step in computer-assisted drug design. In this study, unbiased molecular dynamics simulations have been performed to explain the stability of Indicaxanthin as an inhibitor against the two forms of hIKKβ, active and inactive, and allosteric inhibitor of the inactive form. Additionally, we tried to understand if the protein target undergoes conformational alteration after interacting with Indicaxanthin. Therefore, starting from the previous IFD poses which BPMD analysis showed to be accurate and reliable except for Indicaxanthin in the allosteric site, five systems have been generated and submitted each for 100 ns in MD simulations (Chain A-Indicaxanthin, Chain B-Indicaxanthin, Chain A allosteric site-Indicaxanthin, Chain A-K252a, Chain B-K252a). The various analysis such as the root mean square deviation (RMSD), the root mean square fluctuation (RMSF), number, and types of protein-ligand contacts have been carried to have a more detailed analysis of Indicaxanthin-target complexes compared to the co-crystallized ligand.

#### Stability Analysis

**FIGURE 5:**
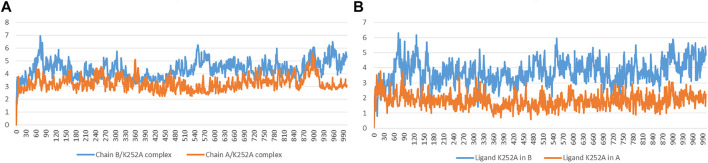
Time evolution of the RMSD (Å) values of backbone atoms **(A)** and ligand K252a **(B)** in the active form (Chain B) and the inactive form (Chain A).

The RMSD has been selected as a criterion to evaluate the dynamic stability of ligand-bound systems. The first systems took into consideration are Chain A and Chain B bound to the co-crystallized inhibitor K252a. We decided to perform these analyses to compare the behavior of Indicaxanthin with respect to an inhibitor with experimental evidence in terms of binding interactions. All protein frames are first aligned on the reference frame backbone, and then the RMSD is calculated based on the atom selection, in these cases on the Cα. For these complexes, the RMSD values of protein’s Cα atoms and ligand are reported in Figure 5. The Ligand RMSD indicates how stable the ligand is with respect to the protein and its binding pocket. This plot shows the RMSD of a ligand when the protein-ligand complex is first aligned on the protein backbone of the reference and then the RMSD of the ligand heavy atoms is measured. If the values observed are significantly larger than the RMSD of the protein, then it is likely that the ligand has diffused away from its initial binding site. The Chain A-K252a system reached equilibrium quickly and fluctuated around the average value of 3 Å, the low average value of ligand RMSD = 1.8 Å indicated strong stability of K252a in the binding pocket as expected due to the low number of rotatable bonds and the eight fused rings. The Chain B-K252a behavior is slightly different. The system reached equilibrium after ∼10 ns and the fluctuation of the protein is higher than the previous system analyzed (∼4.3 Å). The same evidence is reported for the average ligand RMSD that is higher (3.72 Å). This first analysis showed that the phosphorylation of the serine residues in the active form (Chain B) could confer more flexibility to the binding pocket in the KD, as confirmed by the higher fluctuations of K252a despite the rigid structure.

**FIGURE 6:**
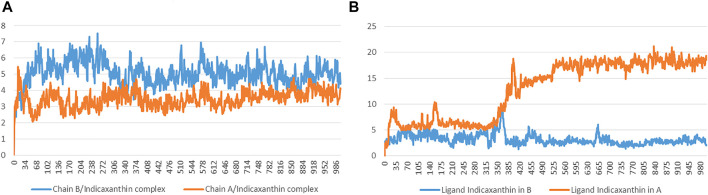
Time evolution of the RMSD (Å) values of backbone atoms **(A)** and ligand Indicaxanthin **(B)** in the active form (Chain B) and the inactive form (Chain A).

For the Chain A-Indicaxanthin and Chain B-Indicaxanthin systems, the RMSD values of protein’s Cα atoms and ligand are reported in Figure 6 showing the behavior of these along the 100 ns of simulations. The Chain A-Indicaxanthin complex, after fluctuating in the first 10 ns, between RMSD values of 2-6Å, reached the equilibrium maintaining the fluctuation around the average value of 3.46Å. But it is worthy to note that ligand RMSD undergoes a biphasic behavior. In fact, in the first 38ns of simulation, the average ligand RMSD value is 6.0 Å, while in the rest of the simulation this value grows about 12 Å, stabilizing around ∼17.5 Å but the ligand continues to occupy the binding pocket acting as a lid. The behavior of the Chain B-Indicaxanthin complex is quite stable if compared to the previously discussed. The system reached rapidly the equilibrium around 7 ns and maintained stable fluctuations along the rest of the simulation with an average RMSD = 5.1 Å. The ligand RMSD, unless a quick cliff around 36–37.5 ns, maintains an average value = 3.2 Å that is similar to the behavior of the co-crystallized K252a. This value is interesting considering the less rigid structure of Indicaxanthin and explaining the good stability of this compound in the KD binding pocket.

For the complex of the Indicaxanthin docked into the allosteric site of Chain A, the protein maintains a stable behavior for the whole duration of simulation with an average RMSD value = 3.5 Å. In contrast, the ligand maintains its stability around the allosteric pocket for about 23 ns, but this stability is not maintained over time, and around 40 ns, Indicaxanthin flies out from the allosteric pocket until the end of the simulation. We repeated the simulations two times: in the second simulation the ligand flies out in the first nanoseconds, and in the third, it remains in the allosteric pocket for about 15 ns to fly out in the next nanoseconds (Supplementary Material). These last findings confirmed the BPDM results in which the unstable binding of Indicaxanthin in the allosteric pocket was retrieved.

#### Residues Mobility Analysis

**FIGURE 7:**
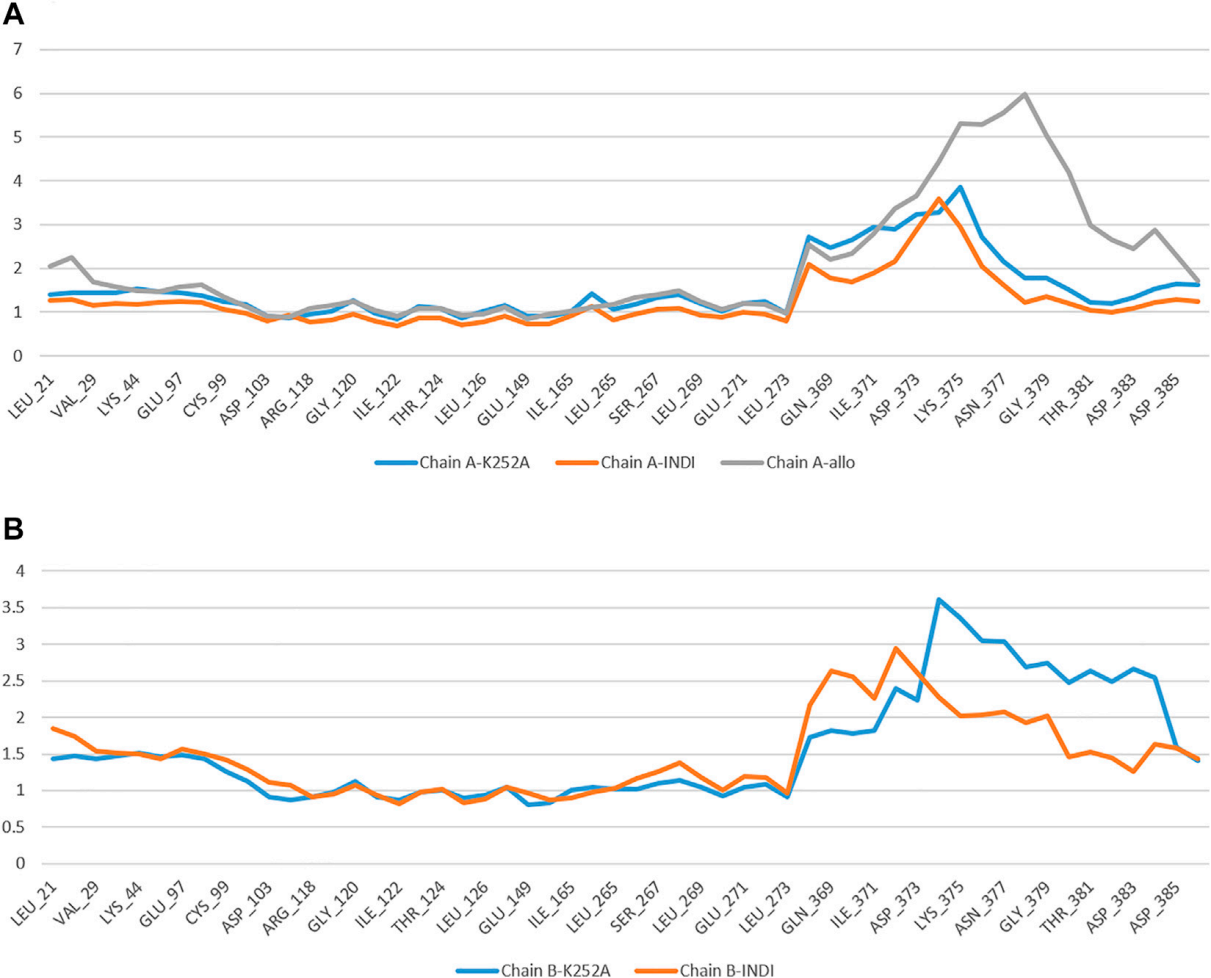
The comparative RMSF values for the complexes of backbone atoms of Chain A **(A)** and Chain B **(B)** bound to K252a, Indicaxanthin, and of the allosteric pocket.

To examine the structural flexibility effect of K252a and Indicaxanthin upon the Chain A, Chain B and the effect of Indicaxanthin when bound to the allosteric site per residue, the main chain average RMSF of the complexes have been calculated for the entire 100ns of simulation. The residue-wise fluctuation of the complexes was plotted and presented in Figure 7. The plot has been coupled according to the chain bound to K252a and Indicaxanthin, respectively, to compare the differences due to different ligands. As reported, the RMSF plot of Chain A is quite comparable for the residues of the active site with ∆RMSF <1 Å. The same could be observed for Chain B in which the ∆RMSF <0.5 Å. The major fluctuations happen in the allosteric pocket where the presence of Indicaxanthin determines an ∆RMSF>5 Å, in particular in the loop Thr368-Lys386. From this RMSF analysis, it can be concluded that the binding of Indicaxanthin determines similar fluctuations of the Chain A and Chain B backbone atoms with respect to K252a, in particular in the residues involved in the binding pocket. Contrariwise, the RMSF of the allosteric pocket is highly influenced by the presence of the Indicaxanthin.

#### Protein-Ligands Contact Analysis

**FIGURE 8:**
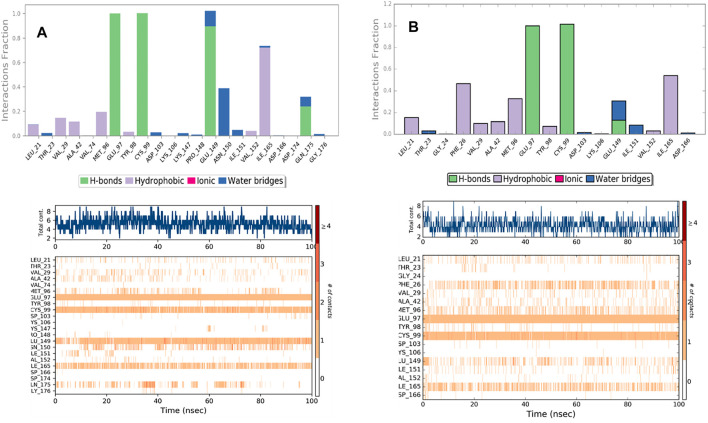
Protein-Ligand contacts: K252a in Chain A **(A)** K252a in Chain B **(B)**.

Estimation of protein interactions provides a measure of interaction power between the ligands and the target protein. Protein interactions with the ligand can be monitored throughout the simulations. These interactions can be categorized by type and summarized, as shown in the plot represented in Figure 8. Protein-ligand interactions (or “contacts’”) are categorized into four types: Hydrogen Bonds, Hydrophobic, Ionic, and Water Bridges. The stacked bar charts are normalized throughout the trajectory: a value of 1.0 suggests that 100% of the simulation time the specific interaction is maintained. A timeline representation of the interactions and contacts (H-bonds, Hydrophobic, Ionic, Water bridges) summarize the total number of specific contacts the protein makes with the ligand throughout the simulation. The bottom panel of Figure 8 shows which residues interact with the ligand in each trajectory frame. Some residues make more than one specific contact with the ligand, which is represented by a darker shade of orange, according to the scale to the right of the plot.

Analyzing the trajectories of Chain A and Chain B with the co-crystallized ligand K252a, it is interesting to note that the residues involved in the interactions are variable both in terms of the type of contacts and in the time of interactions. As reported in Figure 8, K252a has four key residues involved: Met96, Glu97, Cys99, and Ile165 in both active and inactive forms. The H-bonds with Glu97 and Cys99 stable all 100ns simulations long. Hydrophobic contacts of Ile165 are maintained for the major part of the simulations, while hydrophobic contacts with Met96 are involved in both simulations but just for a little fraction of time. The differences in the two complexes regard the less interaction time of the H-bond with Glu149 in Chain B. In Chain A, an H-Bond with Gln175 is observed during the simulation in a discontinuous fashion, while in Chain B, Hydrophobic contacts are observed with Phe26 for about 50% of the simulation time. Other interactions are involved in both complexes such as with Leu21, Thr23, Val29, Ala42, Tyr98, Val152.

**FIGURE 9:**
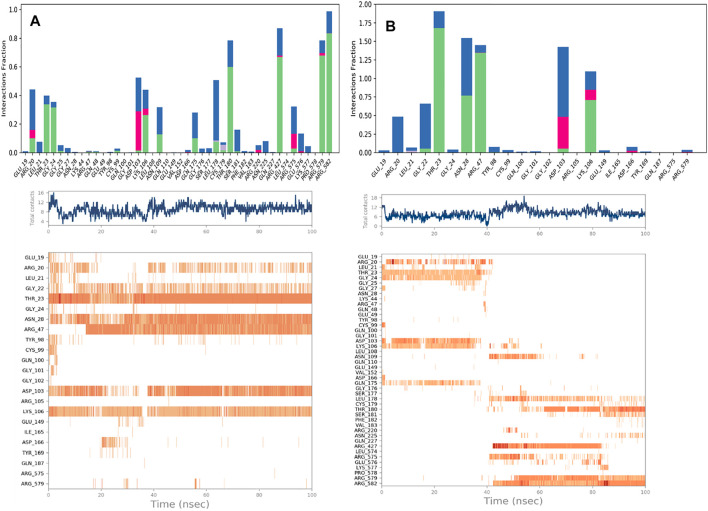
Protein-Ligand contacts: Indicaxantin in Chain A **(A)** Indicaxanthin in Chain B **(B)**. Legenda is the same as Figure 8.

The behavior of Indicaxanthin, when bound to the two forms of IKK, is rather different in terms of interactions, but above in terms of residence in the binding pocket. As previously commented, in Chain A after ∼38 ns Indicaxanthin moves from the deep pocket but remains over it by interacting with H-bonds and ionic interaction with Arg427, Arg575, Arg579, Arg582 acting as a lid. In the first 40ns, the interactions of Indicaxanthin are both ionic and H-bond interactions involving the residues of the pocket such as residues 20–24 and 103–106. Indicaxanthin remains confined into the binding pocket for all the simulation time when Chain B is considered. Even though the residues involved in the protein-ligand interactions are different from those involved in the interaction with K252a. Indicaxanthin interacts for the major of time with Thr23, Asn28, Arg47, and Lys106 through H-bonds, but a key role for the binding stability is due to the ionic interaction with Asp103 and Lys 106 (Figure 9).

The stability of the Indicaxanthin’s binding is also mediated by several water bridges that are hydrogen-bonded protein-ligand interactions mediated by a water molecule. The hydrogen-bond geometry is slightly relaxed from the standard H-bond definition. The current geometric criteria for a protein-water or water-ligand H-bond are a distance of 2.8 Å between the donor and acceptor atoms (D—H···A); a donor angle of 110° between the donor-hydrogen-acceptor atoms (D—H···A); and an acceptor angle of 90° between the hydrogen-acceptor-bonded atoms (H···A—X).

Due to the evidence that during the simulation of Indicaxanthin into the allosteric pocket of Chain A, the ligand flies out after about 40 ns of simulation time, we do not comment on the evolution of the interaction in this analysis. Likely, Indicaxanthin does not act as an allosteric inhibitor of IKK.

### MM-GBSA and Binding Free Energy Analysis

**FIGURE 10:**
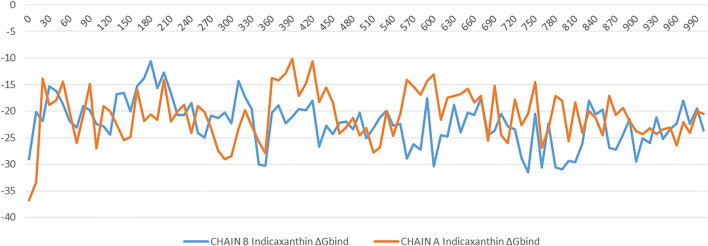
The binding energy (∆G expressed in kcal/mol) landscape of Indicacaxanthin bound to Chain A and Chain B.

To understand the biophysical basis of molecular recognition of Indicaxanthin with targets (the inactive form of IKK, and active form of IKK), a molecular mechanics-generalized Born surface area MM-GBSA approach was used. As a comparative analysis, we performed the binding free energy analysis for the K252a complexes. It provides different individual components such as ∆G_vdW_, ∆G_Coul_, ∆G_Hbond_, ∆G_Lipo_. The conformational entropy change ‒TΔS can be computed by normal-mode analysis on a set of conformational snapshots taken from MD simulations, but many authors have reported that the lack of the evaluation of the entropy is not critical for calculating the MM-GBSA (or MM-PBSA) free energies for similar systems (Hou and Yu, 2007; Massova and Kollman, 2000; Wang and Kollman, 2001). For these reasons, we did not carry out ‒TΔS calculation in our study. For binding free calculation of Indicaxanthin and K252a/protein systems, 101 frames from 100 ns (each 1 ns) were retrieved to calculate ∆Gbind and individual contributions. A summary of binding components in the binding free energy is reported in Table 2. Furthermore, in Figure 10 it is reported the free energy landscape of Indicaxanthin in Chains A and B. To get a better view of which energy terms have more impact on the inhibitory potency, these individual energy components were compared. As expected from previous analysis of the MD trajectories, Indicaxanthin showed a higher binding affinity for the Chain B concerning Chain A (∆G_bind_ = −22.2 ± 4.3 kcal/mol, ∆G_bind_ = −20.7 ± 4.7 kcal/mol, respectively) that it could be justified by the shifting of Indicaxanthin towards the mouth of the binding pocket. It can be seen that ΔG_vdW_ has the major favorable contribution to the total free energy in both complexes. The same consideration could be stated for ΔG_Hbond_ and ΔG_Lipo_, but there is no great difference among these values for different complexes even though it has minor weight on the total free energy. ΔG_Hbond_ has a major impact in Chain A, while ΔG_Lipo_ has a major impact in Chain B. The ΔG_Coul_ has the same effect in the complexes with a negative contribution to the global free energy which is partially balanced by the ∆G_Solv_. As previously stated, Indicaxanthin has been demonstrated to inhibit 50% cell proliferation at 100 µM (Allegra et al., 2018), while K252a showed to be an efficient inhibitor of IKK at IC_50_ = 0.09–018 µM (Sadler et al., 2004). Comparing the binding free energy values of Indicaxanthin and K252a, they are consistent with the experimental evidence (K252a ∆G_bind_ = −72.02 ± 4.1 kcal/mol for Chain B, and ∆G_bind_ = −74.45 ± 4.0 for Chain A) (Table 2).

**TABLE 2:** Predicted MM-GBSA free energies (kcal/mol) and individual energy terms of the Indicaxanthin-target complexes and K252a-target complexes.

	∆G _bind_	∆G _Coul_	∆G _Hbond_	∆G _Lipo_	∆G _Solv_	∆G _vdW_
Indi/Chain B	−22.2 ± 4.3	67.5 ± 11.5	−3.9 ± 1.0	−4.5 ± 0.8	−59.2 ± 11.2	−24.6 ± 4.3
Indi/Chain A	−20.7 ± 4.7	10.6 ± 30.3	−4.3 ± 1.5	−3.4 ± 1.3	−3.2 ± 0.28	−22.3 ± 5.4
K252a/Chain B	−72.02 ± 4.1	−16.78 ± 3.0	−1.33 ± 0.2	−20.62 ± 1.3	24.2 ± 2.1	−57.6 ± 2.5
K252a/Chain A	−74.45 ± 4.0	−22.42 ± 3.3	−1.68 ± 0.3	−18.67 ± 0.3	26.6 ± 1.9	−57.96 ± 2.9

## Conclusion

The stability of Indicaxanthin-hIKKβ complexes compared to K252a, a co-crystallized inhibitor, was assessed by using Induced fit docking, binding pose metadynamics, and molecular dynamics. Finally, MM-GBSA free energy calculations have been performed to establish what form of IKK Indicaxanthin prefers. Induced fit docking results showed that the binding of Indicaxanthin with the active form, the inactive form, and the allosteric site of hIKKβ showed has the strongest stability with the active form. MD trajectories analysis (RMSD, RMSF, and protein-ligand contacts number and along the time) also showed that Indicaxanthin enhanced the stability of the active form at the same level of the known inhibitor K252a. The stability of the inactive form complex with Indicaxanthin is quite similar but it did not reach the quality of the active form. Contrariwise, even though for 40 ns over 100 nsIndicaxanthin can bind the allosteric pocket, it should not be considered as an allosteric inhibitor of hIKKβ.

As a whole, this work shows that Indicaxanthin is able, *in silico*, to inhibit the active form of the hIKKβ and adds novel mechanistic insights on its recently discovered ability to impair NF-κB signaling in melanoma A375 cells. Along these lines, present results further suggest the molecule as a useful nutraceutical tool in combo-therapy i.e., with other therapeutical agents targeting different checkpoints of melanoma development.

The currently demonstrated ability of Indicaxanthin to inhibit the active form of hIKKβ may, then, suggest the phytochemical as a new lead compound to synthesize novel and more potent IKKβ inhibitors for the treatment of cancer and inflammation-related conditions.

## Data Availability

The raw data supporting the conclusions of this article will be made available by the authors, without undue reservation.
