# Supplementary material for: Evaluation of the IKKβ Binding of Indicaxanthin by Induced-Fit Docking, Binding Pose Metadynamics, and Molecular Dynamics
Source: Front Pharmacol. 2021 Sep 10;12:701568. doi: 10.3389/fphar.2021.701568 (PMC8461089; doi:10.3389/fphar.2021.701568)
Supplement: Supplementary file 1 [file DataSheet1.PDF]

Evaluation of the IKKβ binding of Indicaxanthin by induced-fit docking, binding pose metadynamics, and molecular dynamics

Mario Allegra[1], Marco Tutone[1*], Luisa Tesoriere[1], Alessandro Attanzio[1], Giulia Culletta[1], Anna Maria Almerico[1]

1 Dipartimento di Scienze e Tecnologie Biologiche Chimiche e Farmaceutiche (STEBICEF), Università degli Studi di Palermo, Via Archirafi 28, 90123 Palermo, Italy

*** Correspondence:**
Marco Tutone
marco.tutone@unipa.it

Supplementary Infomation

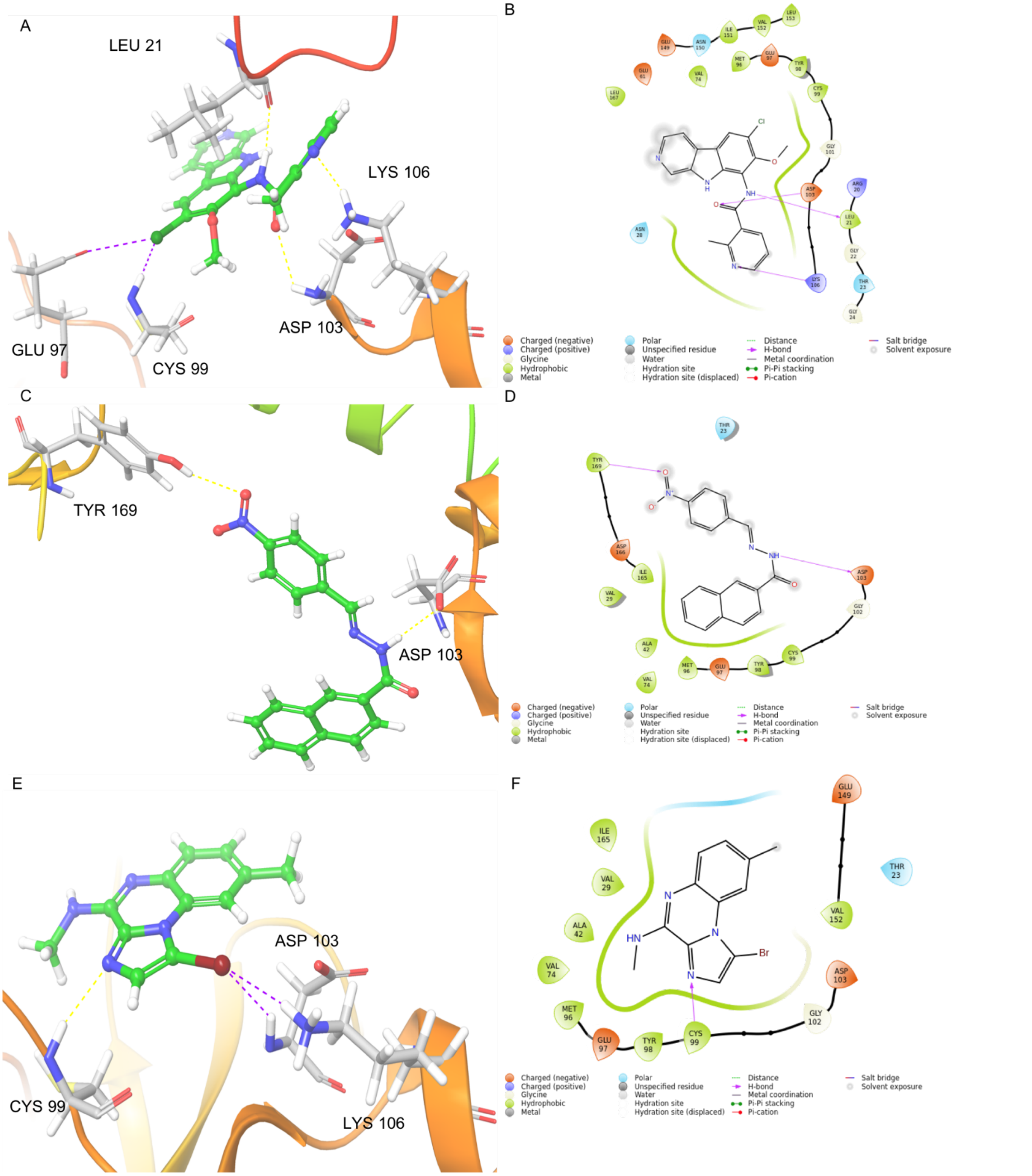

Figure SI1. A) 3D pose of MLN120B in the active Chain B of hIKKβ; B) 2D pose of MLN120B in the active Chain B of hIKK β; C) 3D pose of the imidazo[12-a]quinoxaline derivative 6a in the active Chain B of hIKKβ; D) 2D pose of the imidazo[12-a]quinoxaline derivative 6a in the active Chain B of hIKKβ; E) 3D pose of the LASSBio-1524 in the active Chain B of hIKKβ; F) 2D pose of the the LASSBio-1524 in the active Chain B of hIKKβ

In the 3D figures, H-bonds interactions are represented in yellow dashes, the halogen-H bonds in purple dashes

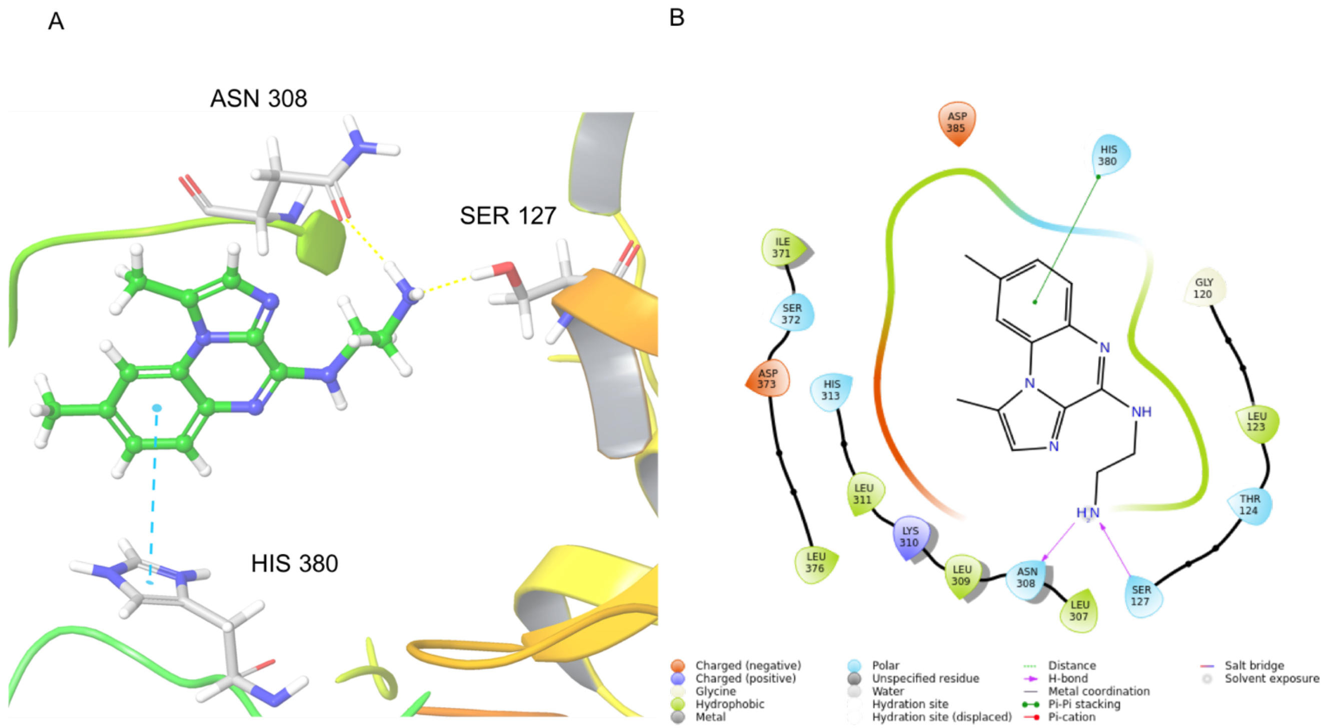

Figure SI2. A) 3D pose of BMS345521 in the allosteric site of hIKKβ; B) 2D pose of BMS345521 in the allosteric site of hIKKβ

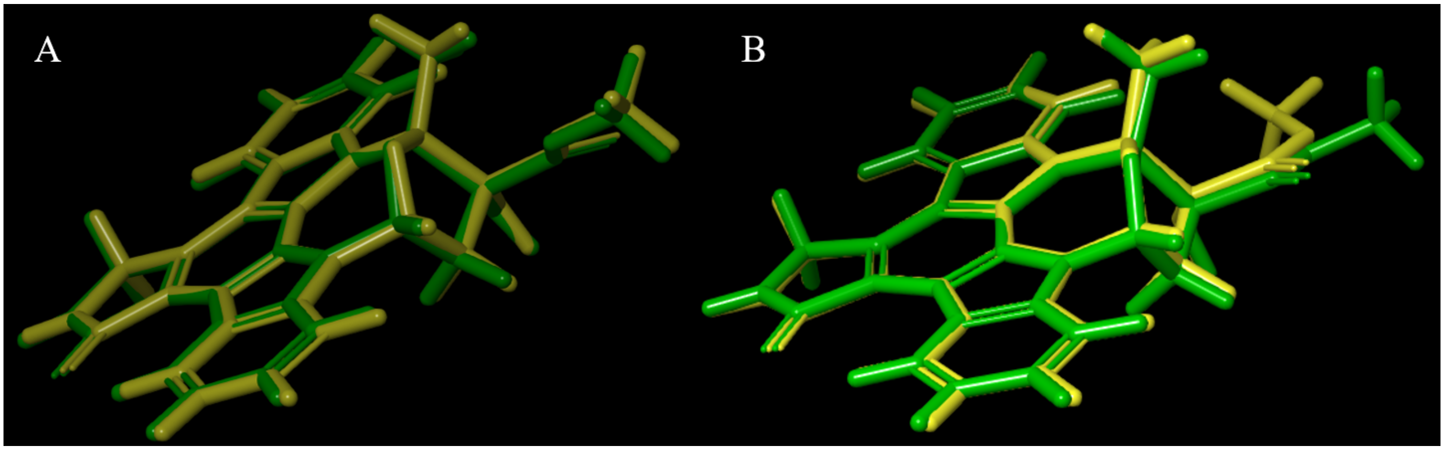

Figure SI3. A) Re-docking of K252a in Chain A, in green the experimental pose, in yellow the docked pose; B) Re-docking of K252a in Chain B, in green the experimental pose, in yellow the docked pose

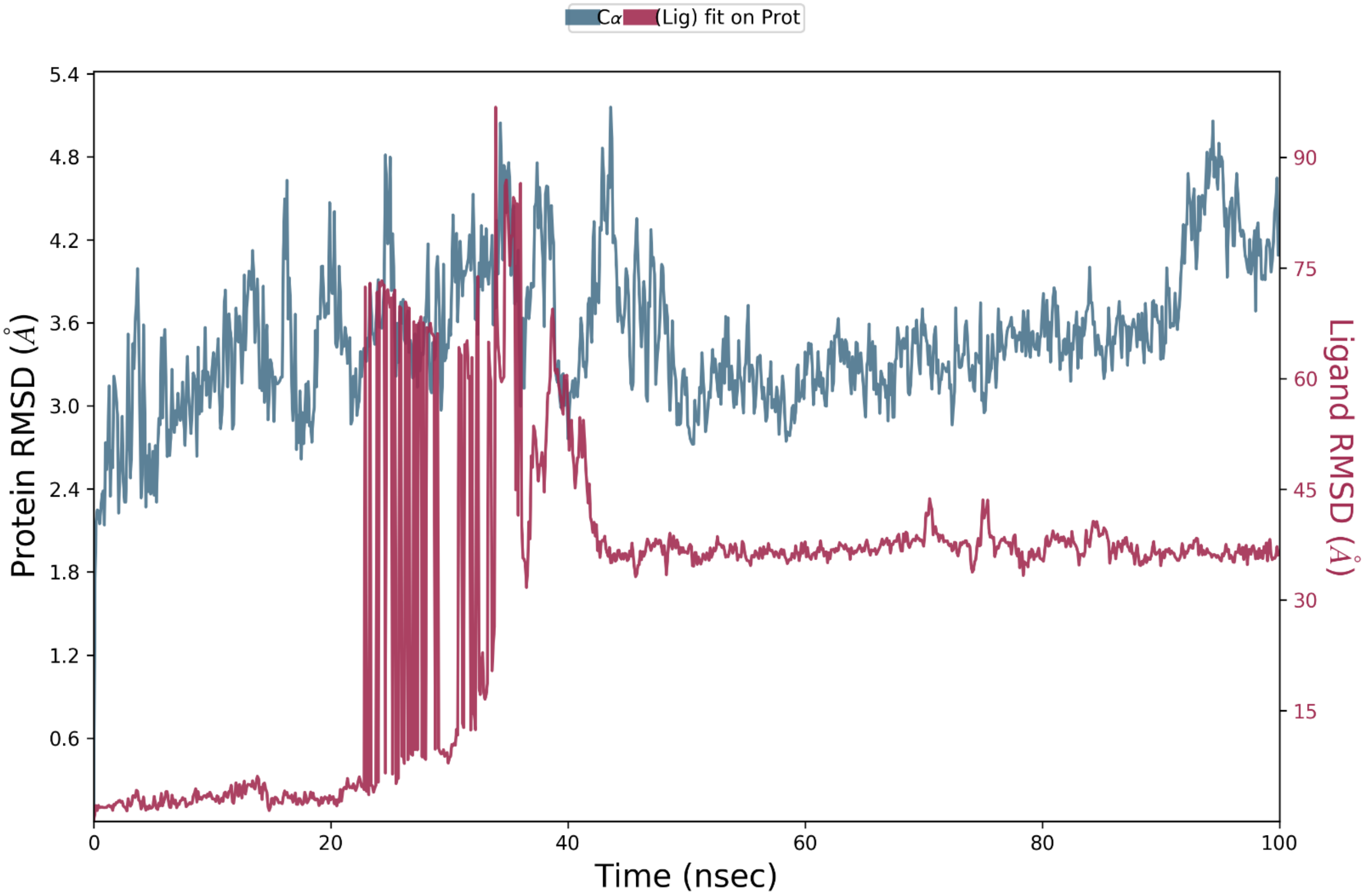

Figure SI4. 1st simulation of Indicaxanthin into the allosteric pocket

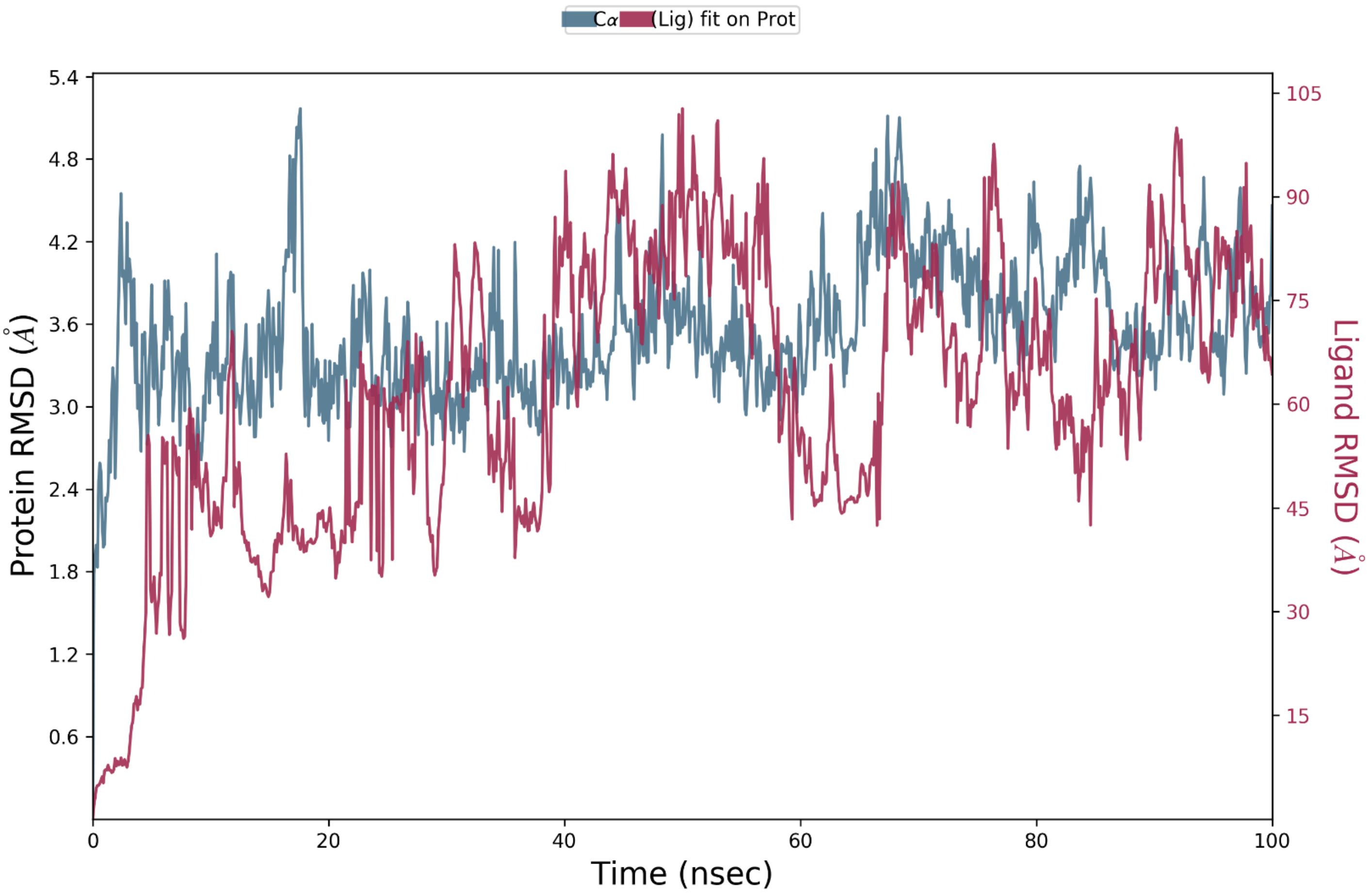

Figure SI5. 2nd simulation of Indicaxanthin into the allosteric pocket

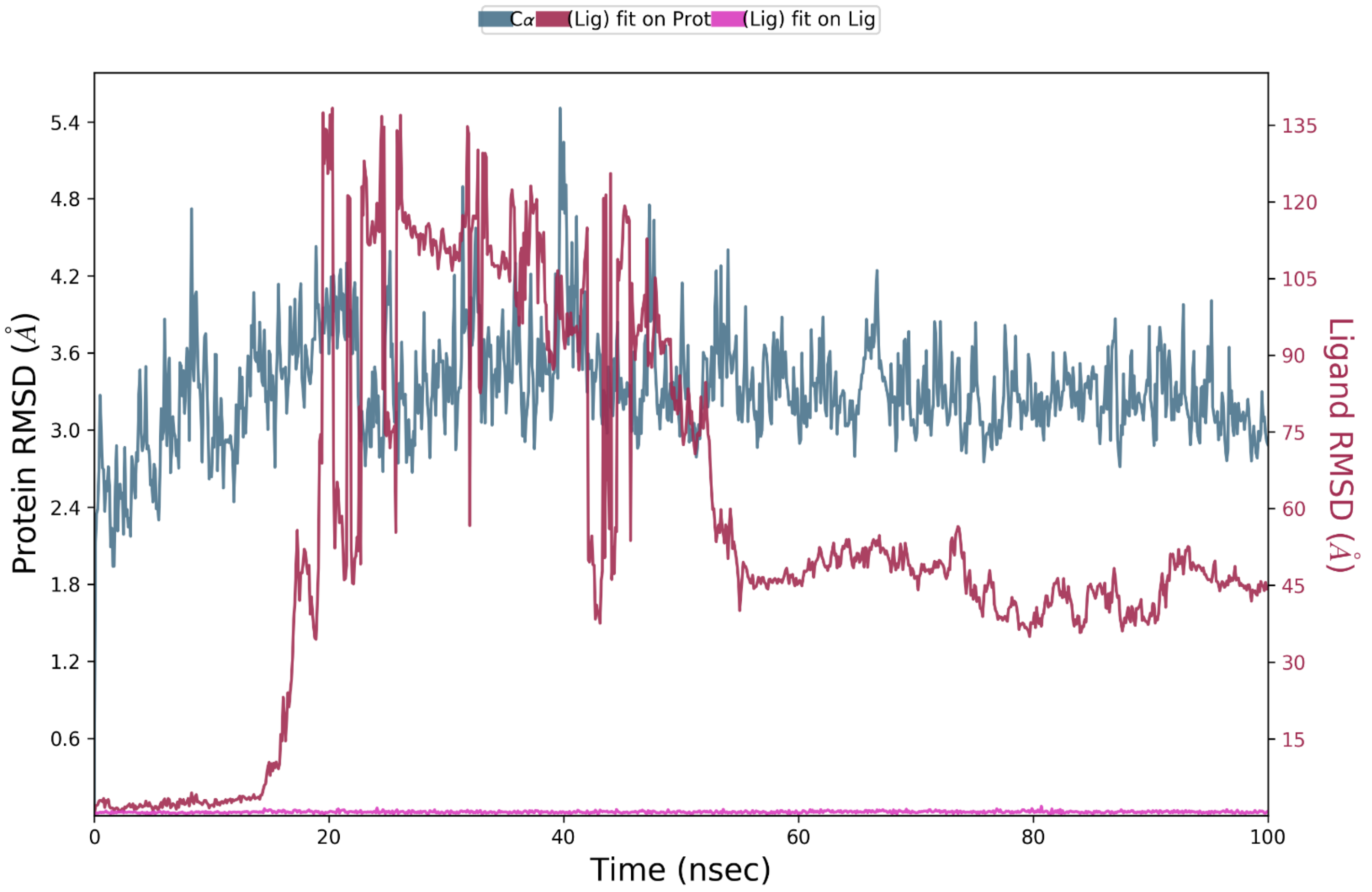

Figure SI6. 3rd simulation of Indicaxanthin into the allosteric pocket

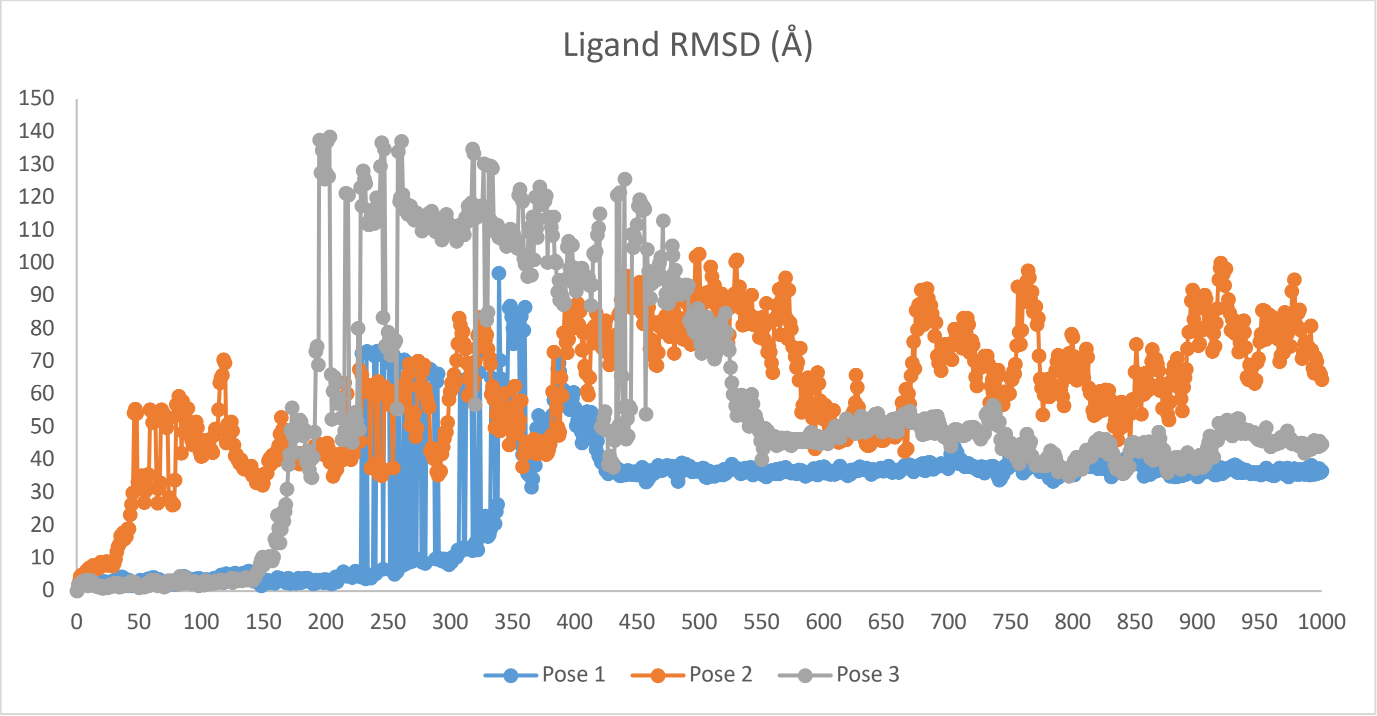

Figure SI7. Compared Indicaxanthin RMSD into the allosteric pocket

	Compound	Residue	Interaction	Docking Score	IC_{50}
Orthosteric	MLN120B	Leu 21	H bond with NH amide	-10.253	60nm
		Asp 103	H bond with CO		
		Lys 106	H bond with N of 2-methyl-pyridine		
		Glu 97	Halogen bond with Cl		
		Cys 99	Halogen bond with Cl		
	6a	Cys 99	H bond with N of 3-bromo-imidazole	-7.617	324nm
		Asp 103	Halogen-H bond with Br		
		Lys 106	Halogen-H bond with Br		
	LASSBIO1524	Asp 103	H bond with NH amide	-7.230	20um
		Tyr 169	H bond with NO		
Allosteric	BMS345521	His 380	pi stacking with the fused toluyl ring	-7.730	300nm
		Asn 308	H bond with the terminal amino group		
		Ser 127	H bond with the terminal amino group		

Table S1. Docked reference inhibitors
